# Analysis of significant protein abundance from multiple reaction-monitoring data

**DOI:** 10.1186/s12918-018-0656-9

**Published:** 2018-12-31

**Authors:** Jongsu Jun, Jungsoo Gim, Yongkang Kim, Hyunsoo Kim, Su Jong Yu, Injun Yeo, Jiyoung Park, Jeong-Ju Yoo, Young Youn Cho, Dong Hyeon Lee, Eun Ju Cho, Jeong-Hoon Lee, Yoon Jun Kim, Seungyeoun Lee, Jung-Hwan Yoon, Youngsoo Kim, Taesung Park

**Affiliations:** 1 Department of Statistics, Seoul National University, Seoul, South Korea; 2 Graduate School of Public Health, Seoul National University, Seoul, South Korea; 3 Department of Biomedical Engineering, Seoul National University College of Medicine, Seoul, South Korea; 4 Institute of Medical and Biological Engineering, Medical Research Center, Seoul National University College of Medicine, Seoul, South Korea; 5 Department of Internal Medicine and Liver Research Institute, Seoul National University, Seoul, South Korea; 6 Department of Mathematics and Statistics, Sejong University, Seoul, South Korea; 7 Interdisciplinary program in Bioinformatics, Seoul National University, Seoul, South Korea

**Keywords:** Multiple reaction-monitoring (MRM), Protein, Logistic regression-based method for significance analysis of multiple reaction monitoring (LR-SAM), Hepatocellular carcinoma (HCC), Sorafenib response

## Abstract

**Background:**

Discovering reliable protein biomarkers is one of the most important issues in biomedical research. The ELISA is a traditional technique for accurate quantitation of well-known proteins. Recently, the multiple reaction-monitoring (MRM) mass spectrometry has been proposed for quantifying newly discovered protein and has become a popular alternative to ELISA. For the MRM data analysis, linear mixed modeling (LMM) has been used to analyze MRM data. MSstats is one of the most widely used tools for MRM data analysis that is based on the LMMs. However, LMMs often provide various significance results, depending on model specification. Sometimes it would be difficult to specify a correct LMM method for the analysis of MRM data. Here, we propose a new logistic regression-based method for Significance Analysis of Multiple Reaction Monitoring (LR-SAM).

**Results:**

Through simulation studies, we demonstrate that LMM methods may not preserve type I error, thus yielding high false- positive errors, depending on how random effects are specified. Our simulation study also shows that the LR-SAM approach performs similarly well as LMM approaches, in most cases. However, LR-SAM performs better than the LMMs, particularly when the effects sizes of peptides from the same protein are heterogeneous. Our proposed method was applied to MRM data for identification of proteins associated with clinical responses of treatment of 115 hepatocellular carcinoma (HCC) patients with the tyrosine kinase inhibitor sorafenib. Of 124 candidate proteins, LMM approaches provided 6 results varying in significance, while LR-SAM, by contrast, yielded 18 significant results that were quite reproducibly consistent.

**Conclusion:**

As exemplified by an application to HCC data set, LR-SAM more effectively identified proteins associated with clinical responses of treatment than LMM did.

## Background

Discovering protein disease biomarkers is an urgently pressing issue in biomedical research [1]. Historically, the enzyme-linked immunosorbent assay (ELISA) is a highly accurate protein quantitation technique [2], representing the “gold standard” for measuring levels of specific proteins [3]. However, recent discoveries of many novel proteins, having no available antibodies, now limits the use of the ELISA method [4]. Moreover, for newly discovered proteins, development of high quality ELISA assays requires considerable time and resources [5]. Together, these shortcomings have created a need for a different technique of targeted protein quantitation [6]. One such technique, quantitative mass spectrometry of proteins has advanced significantly over the last decade, and several methods have been developed for relative quantification of targeted proteins, using this technique [7].

In recent years, multiple reaction monitoring (MRM) mass spectrometry has been developed as an attractive tool for targeted proteins and now represents a promising alternative to ELISA for quantification of proteins. To that end, MRM uses sequence-specific tandem mass spectrometer fragmentations of peptides that can provide highly selective measurements for peptide-containing proteins [8]. Thus, without enrichment or fractionation approaches, MRM assays allow for the quantitation of protein ions within ranges of low (μg/mL) to high (ng/mL) levels [5, 9]. Additionally, development time for MRM assays is relatively shorter and less expensive than that for ELISA, with no requirements (and thus no costs) for antibody development.

Due to these many advantages, MRM assays are being increasingly used in systems biology and clinical investigations [10–13]. There are some analysis tools to analyze MRM data. Skyline is representative tool to create and analyze MRM dataset [14]. ProteoSign gives some simple statistical analysis results to find differentially expressed proteins [15]. roHits-viz is a web-based tool for visualizing interaction of protein data [16]. However, the development of statistical methods to determine significantly abundant proteins by the MRM assay has not received enough attention, compared to improvement of the MRM assay technology itself [17]. For MRM data analysis, two-sample t-tests or paired t-tests have been applied to identify proteins that change in abundance between two groups [18–20]. To test for multiple groups, one-way analysis of variance (ANOVA) has been employed [21, 22].

Recently, a linear mixed model (LMM) approach was proposed for MRM data analysis, and was implemented in MSstats (v3.7.1) [17], resulting in its widespread adoption [23]. LMM approaches include a single parameter for a group effect, peptide effects, and group and peptide interaction effects, along with subject and run effects. LMMs include both subject and run effects that can be designated as either random or fixed, to identify significantly evident proteins by testing the group effect. Recently, we observed that the LMM approach often provides different *p*-values for the group effect, from the same data, depending on which effects are treated as random or fixed. In particularly, the LMM test results vary considerably when there is an interaction effect between group and peptide.

In this report, we propose a new logistic regression-based method for Significance Analysis of Multiple Reaction Monitoring (LR-SAM). Unlike LMMs, our LR-SAM approach uses a much smaller number of parameters. Moreover, our LR-SAM does not require inclusion of all the effects related to the run. Accordingly, our model does not need to specify run effects as random or fixed.

Since LR-SAM uses log2-transformed relative intensity values, it does not need to include run effects. We consider two separate cases when the effects of peptides in a protein are homogeneous or heterogeneous. For the significance test for proteins, we consider Wald type tests, likelihood ratio tests (LRTs), and score tests [24].

Through various simulation studies, we compared the performance of LMMs to our approach. We first observed cases in which an LMM approach did not preserve type I error, and we also examined cases in which LR-SAM methods performed better. Some tests, based on the LR-SAM method, were more powerful when the expression pattern of peptide between groups was heterogeneous.

To further establish the translational relevance of our method, we compared LMMs to our approaches in a clinical study to identify candidate serum biomarkers of sorafenib response and prognosis in patients with hepatocellular carcinoma (HCC). Sorafenib is a multikinase inhibitor mainly used for the treatment of various solid tumors including HCC [25]. However, the response rate of sorafenib is about 8% for HCC patients. Due to its high cost, it would be more economical and more beneficial to patients if Sorafenib is applied only to the patients with high chance of response. Moreover, there are no good markers to predict the patients’ response to sorafenib [26]. From May 2013 to August 2014, 115 HCC patient serum samples were collected from Seoul National University Hospital as part of an ongoing HCC study. One hundred twenty-four candidate protein biomarkers, and hepatic disease-associated proteins, were chosen from 50,265 proteins, based on the LiverAtlas database [27]. Sorafenib responses were measured according to the modified response evaluation criteria in solid tumors (mRECIST) guidelines [28]. Patients with complete responses, partial responses, or stable disease were categorized as responders, while those with progressive disease were categorized non-responders. Both LMM approaches and LR-SAM methods were employed to analyze the MRM data.

## Methods

### MRM dataset structure

Table 1 shows the structure of MRM dataset. Sample ID indicates individual, Run the shared run membership of the endogenous, and Area Ratio the expression of peptide detected. The MRM data has a hierarchical structure such that one protein contains several peptides. Since our goal is to identify proteins that are significantly different between the group, we need to combine the summarized expression of peptides from the same protein efficiently. Also, the batch effect would occur when MRM experiments were performed in different batches separately. These characteristics of MRM make it difficult to use the standard analyses such as two sample t-test.

**Table 1:** MRM dataset structure

Sample ID	Group	Run	Protein	Peptide	Area Ratio
1	1	1	X1	1	*y*_11(1)11_
1	1	1	X1	2	*y*_11(1)21_
2	2	1	X1	1	*y*_22(2)11_
2	2	1	X1	2	*y*_22(2)21_

### Review of LMM approach

For a given protein, suppose it is comprised of K peptides. Let ***y***_***i***, ***j***(***i***), ***k***, ***l***_ denote the log2(intensity) value of the j-th subject, nested in the i-th group of the k-th peptide and the l-th run. Then, the LMM used in MSstats is given as follows:

1 $$ {\mathrm{y}}_{\mathrm{i},\mathrm{j}\left(\mathrm{i}\right),\mathrm{k},\mathrm{l}}=\mu +{G}_i+S{(G)}_{j(i)}+{P}_k+{R}_l+{\left(\mathrm{G}\times \mathrm{P}\right)}_{\mathrm{i},\mathrm{k}}+{\left(P\times R\right)}_{k,l}+{\epsilon}_{i,j(i),k,l}, $$

where *μ* is the global mean; *G*_*i*_ is the i-th group effect; *S*(*G*)_*j*(*i*)_ representing the j-th subject effect nested in the i-th group; *P*_*k*_ stands for the k-th peptide effect; *R*_*l*_ stands for the l-th run effect, (*G* × *P*)_*ik*_ stands for the interaction effect between the i-th group and the k-the peptide; and (*P* × *R*)_*kl*_ signifies the interaction effect between the k-th peptide and the l-th run. When all the effects are treated as fixed, these parameters have the following restrictions: $\sum \limits_{i=0}^2{G}_i=0$, $\sum \limits_{j(i)=1}^{J(i)}S{(G)}_{j(i)}=0$, $\sum \limits_{k=1}^K{P}_k=0$, $\sum \limits_{l=1}^L{R}_l=0$, $\sum \limits_{i=0}^2{\left(G\times P\right)}_{i,k}=0$, $\sum \limits_{k=1}^K{\left(G\times P\right)}_{i,k}=0$, $\sum \limits_{k=1}^K{\left(P\times R\right)}_{k,l}=0,\sum \limits_{l=1}^L{\left(P\times R\right)}_{k,l}=0$, and ${\epsilon}_{i,j(i),k,l}\sim N\left(0,{\sigma}_{\epsilon}^2\right)$. Here, *G*_0_ stands for the effect of a reference group of MRM data. When the subject and run effects are treated as random, the restrictions on *S*(*G*)_*j*(*i*)_, *R*_*l*_, and (*P* × *R*)_*kl*_ are replaced by $S{(G)}_{j(i)}\sim N\left(0,{\sigma}_S^2\right)$, ${R}_l\sim N\left(0,{\sigma}_R^2\right)$ and ${\left(P\times R\right)}_{kl}\sim N\left(0,{\sigma}_{P\times R}^2\right)$, respectively.

For most MRM data analyses, the investigator’s interest lies in determining which proteins differ in abundance between two groups. Thus, the hypothesis of interest is given below for comparing two groups:

2 $$ {H}_0:{G}_1={G}_2,{\mathrm{H}}_1:{\mathrm{G}}_1\ne {\mathrm{G}}_2 $$

The MSstats uses the t-test for this hypothesis.

### LR-SAM approach

Our proposed LR-SAM approach uses a log2-transformed relative intensity value instead of the original log2-transformed intensity value itself. A log2-transformed relative intensity value was derived as ***y***_***i***, ***j***(***i***), ***k***, ***l***_ − ***y***_**0**, **0**(**0**), ***k***, ***l***_. In that scenario, ***P***_***k***_, ***R***_***l***_ and (***P*** × ***R***)_***kl***_ effects that share the same k and l values are removed. If we denote ***y***_***j***, ***k***_ as a log2-transformed relative intensity value of the j-th subject and the k-th peptide, where j = 1,J(1),J(1) + 1,…,J(1) + J(2). Then, the log2-transformed relative intensity value ***y***_***j***, ***k***_ stands for the ***y***_**1**, ***j***(**1**), ***k***, ***l***_ − ***y***_**0**, **0**(**0**), ***k***, ***l***_ for j = 1,…,J(1), and it stands for the ***y***_**2**, ***j***(**2**), ***k***, ***l***_ − ***y***_**0**, **0**(**0**), ***k***, ***l***_ for j = J(1) + 1,…,J(1) + J(2).

#### LR-SAM with fixed effect

Consider a logistic regression model using log2-transformed relative intensity value, as follows:

3 $$ logit\left(P\left({Z}_j=1\right)\right)=\alpha +{\beta}_1{y}_{j,1}+\dots +{\beta}_K{y}_{j,K}, $$

where *Z*_*j*_ is a group indicator of the j-th subject that is assumed to follow a Bernoulli distribution; *α* is an intercept; and *β*_*k*_ is the coefficient of the k-th peptide. Note that the *β*_*k*_ values are related to *G*_1_ − *G*_2_ + (*G* × *P*)_1, *k*_ − (*G* × *P*)_2, *k*_ of model (1). Therefore, if we treat all *β*_*k*_’s as fixed, the hypothesis for comparing the two groups is:

4 $$ {H}_0:{\beta}_1=\cdots ={\beta}_K=0 $$

To test (4), we consider the likelihood ratio test (L) with *K* degrees of freedom, as follows:

5 $$ \mathrm{L}=-2\left({l}_0\left({\widehat{\alpha}}_0\right)-l\left(\widehat{\alpha},{\widehat{\beta}}_1,\dots, {\widehat{\beta}}_K\right)\right) $$

Here, ${\widehat{\alpha}}_0$ is the maximum likelihood estimate (MLE) of *α* under the null hypothesis (see above). ${l}_0\left({\widehat{\alpha}}_0\right)$ is the maximum likelihood value under the null hypothesis, and $\widehat{\alpha},{\widehat{\beta}}_1,\dots, {\widehat{\beta}}_K$ are the MLEs of *α*, *β*_1_, …, *β*_*K*_, respectively. It is also known that L asymptotically follows a chi-square distribution, with *K* degrees of freedom.

A Wald type test (W) statistic for analysis, is given below:

6 $$ W={\left(\begin{array}{c}{\widehat{\beta}}_1\\ {}\vdots \\ {}{\widehat{\beta}}_K\end{array}\right)}^{\prime } Var{\left({\widehat{\beta}}_1,\dots, {\widehat{\beta}}_K\right)}^{-1}\left(\begin{array}{c}{\widehat{\beta}}_1\\ {}\vdots \\ {}{\widehat{\beta}}_K\end{array}\right) $$

Here, *W* also asymptotically follows a chi-square distribution, with *K* degrees of freedom under the null hypothesis. If we assume that the *β*_*k*_ values are homogeneous, a Wald test with 1 degree of freedom (*W*_1_) can be considered as follows:

7 $$ {W}_1={\widehat{\beta}}_p{A}^{-1}{\widehat{\beta}}_p, $$

in which ${\widehat{\beta}}_p$ is the weighted average of ${\widehat{\beta}}_k$ as $\sum \limits_{k=1}^K{t}_k{\widehat{\beta}}_k$, where *t*_*k*_ is a weight given as $\frac{1/ Var\left({\widehat{\beta}}_k\right)}{\sum_{k=1}^K1/ Var\left({\widehat{\beta}}_k\right)}$, and *A* is the variance of ${\widehat{\beta}}_p$, given as $$ {\left(\begin{array}{c}{t}_1\\ {}\vdots \\ {}{t}_K\end{array}\right)}^{\prime } Var\left({\widehat{\beta}}_1,\dots, {\widehat{\beta}}_K\right)\left(\begin{array}{c}{t}_1\\ {}\vdots \\ {}{t}_K\end{array}\right) $$. Thus, *W*_1_ asymptotically follows a chi-square distribution, with 1 degree of freedom, under the null hypothesis. Moreover, if the *β*_*k*_values are homogeneous, Eq. (3) (shown above) can be reduced to the following model:

8 $$ logit\left(P\left({Z}_j=1\right)\right)=\alpha +{\beta}^{\ast}\sum \limits_{k=1}^K{y}_{j,k} $$

The hypothesis of interest from this model (8) is *H*_0_ : *β*^∗^ = 0, and a Wald test (*W*_*S*_) is given by:

9 $$ {W}_S={\widehat{\beta}}^{\ast }\  Var{\left({\widehat{\beta}}^{\ast}\right)}^{-1}\ {\widehat{\beta}}^{\ast } $$

Here, ${\widehat{\beta}}^{\ast }$ is the maximum likelihood estimate of *β*^∗^ from the model (8), and *W*_*S*_ asymptotically follows a chi-square distribution, with 1 degree of freedom.

#### LR-SAM with random effects

When effects of peptides were heterogeneous, we could detect significant heterogenous effect by assuming random effects on coefficients β_k_. This assumption is commonly used in meta-analysis or rare variant analysis of genetic study [24, 29]. If we assume that *β*_*k*_ follows a normal distribution, with mean 0 and variance *w*_*k*_*τ* (where *w*_*k*_ is a known prior weight), then the logistic regression model (3) is expanded to a mixed effect model, and the hypothesis (4) is equivalent to:

10 $$ {H}_0:\tau =0,{H}_1:\uptau \ne 0 $$

Since the hypothesis *H*_0_ : *τ* = 0 is on the boundary of the parameter space, the variance-component score test can be considered. The score test statistic of the variance-component for (10) is:

11 $$ {S}_{VC}={\left(\boldsymbol{Z}-{\widehat{\boldsymbol{\mu}}}_0\right)}^{\prime}\boldsymbol{K}\left(\boldsymbol{Z}-{\widehat{\boldsymbol{\mu}}}_0\right) $$

Here, ${\widehat{\boldsymbol{\mu}}}_0$ is the estimated probability under *H*_0_; ***K*** = ***YWY***^′^, where ***Y*** = [***Y***_1_, …, ***Y***_*K*_] and ***Y***_*k*_ = (*y*_1*k*_, …, *y*_*nk*_)^′^; ***Z*** means the group indicator vector, and ***W*** is a diagonal matrix with the k-th element as *w*_*k*_.

It is known that *S*_*VC*_ follows a mixture of chi-square distributions ${\sum}_{k=1}^K{\uplambda}_k{\chi_{1,k}}^2$, where *χ*_1, *k*_^2^’s are independent chi-square distributions with 1 degree of freedom, and λ_*k*_ is the k-th eigenvalue of ***P***^**1**/**2**^***KP***^**1**/**2**^ [24]. Here, $\boldsymbol{P}={\widehat{\boldsymbol{V}}}^{-\mathbf{1}}-{\widehat{\boldsymbol{V}}}^{-\mathbf{1}}\mathbf{1}\left({\mathbf{1}}^{\prime }{\widehat{\boldsymbol{V}}}^{-\mathbf{1}}\mathbf{1}\right){\mathbf{1}}^{\prime }{\widehat{\boldsymbol{V}}}^{-\mathbf{1}}$, where $\widehat{\boldsymbol{V}}$ is a diagonal matrix with the k-th element as ${\widehat{\mu}}_{0k}\left(1-{\widehat{\mu}}_{0k}\right)$. For simplicity, we assume a flat prior weight given by *w*_*k*_ = 1 for *k* = 1, …, *K*.

### Simulation design

We next performed simulation studies to investigate the power of the LMM and LR-SAM approaches for comparing two groups, and whether they preserve type I error. For analysis, there were four LMMs available, depending on how the random or fixed effects were specified: (i) LMM(FF), with fixed subject and run effects, (ii) LMM(FR), with fixed subject effect and random run effects, (iii) LMM(RF), with random subject and fixed run effects, and (iv) LMM(RR), with random subject and run effects. For each simulated dataset, the best LMM, LMM(best), was selected among four LMMs with the smallest Akaike information criterion (AIC) value [30]. Thus, there were five LR-SAM test statistics, **L**, **W**, ***W***_**1**_, ***W***_***S***_, and ***S***_***VC***_, for comparison.

Simulation data was generated from the model, using 1000 repetitions. The global mean, *μ*, was arbitrarily set to 15, while the reference effects, *G*_0_, *S*(*G*)_0, (0)_, and (*G* × *P*)_0, *k*_, for *k* = 1, …, *K*, were set to 0. The normal distribution, with mean 0 and variance 0.5, was set as the error distribution, and the number of peptides was set to 4. For the random subject effect, we generated *S*(*G*)_*j*(*i*)_ from the identical normal distribution independently, with mean 0 and variance 0.25 for *i* = 1, 2. For the fixed subject effect, we set *S*(*G*)_*j*(*i*)_ as follows:

$$ S{(G)}_{j(i)}=-{e}_S+\frac{2\left(j(i)-1\right)}{J(i)-1}{e}_S\ \mathrm{for}\kern0.5em j(i)=1,\dots, J(i)\kern0.75em \mathrm{and}\kern0.5em i=1,2,\mathrm{where}\kern0.5em {e}_S>0\ \kern0.5em \mathrm{and}\kern0.5em {e}_S=\sqrt{3\frac{J(i)-1}{J(i)+1}}{\sigma}_S,\kern0.5em \mathrm{with}\kern0.5em {\sigma}_S^2=0.25. $$

The peptide effect, *P*_*k*_, was considered as a fixed effect, and was set as follows:

$$ {P}_k=-{e}_P+\frac{2\left(k-1\right)}{K-1}{e}_P\kern0.5em \mathrm{for}\kern0.5em k=1,\dots, K,\kern0.5em \mathrm{where}\kern0.5em {e}_P>0\kern0.5em \mathrm{and}\kern0.5em {e}_P=\sqrt{3\frac{K-1}{K+1}}{\sigma}_P\kern0.5em \mathrm{with}\kern0.5em {\sigma}_P^2=0.1 $$

For the random run effect, we generated *R*_*l*_ from the identical normal distribution, independently, with mean 0 and variance 0.25. For the fixed run effect, we set *R*_*l*_ as follows:

$$ {R}_l=-{e}_R+\frac{2\left(m-1\right)}{M-1}{e}_R\kern0.5em \mathrm{for}\kern0.5em m=1,\dots, M,\kern0.5em \mathrm{where}\kern0.5em {e}_R>0\kern0.5em \mathrm{and}\kern0.5em {e}_R=\sqrt{3\frac{M-1}{M+1}}{\sigma}_R,\kern0.5em \mathrm{with}\kern0.5em {\sigma}_R^2=0.25 $$

We then considered the peptide×run interaction effect as either random or fixed, followed by the type of run effect. For the random peptide by run interaction effect, we generated (*P* × *R*)_*k*, *l*_ from the identical normal distribution independently, with mean 0 and variance 0.1. For the fixed peptide×run interaction effect, we set (*P* × *R*)_*k*, *l*_ as follows:

$$ {\left(P\times R\right)}_{k,l}=\left({e}_{PR}-\frac{2\left(k-1\right)}{K-1}{e}_{PR}\right){\left(-1\right)}^l\kern0.5em \mathrm{for}\kern0.5em l=1,\dots, L\kern0.5em \mathrm{and}\kern0.5em k=1,\dots, K,\kern0.5em \mathrm{where}\kern0.75em {e}_{PR}>0\kern0.5em \mathrm{and}\kern0.5em {e}_{PR}=\sqrt{3\frac{K-1}{K+1}}{\sigma}_{PR},\kern0.5em \mathrm{with}\kern0.5em {\sigma}_{PR}^2=0.1 $$

Provided those parameters, we further considered four group effect scenarios (GSs), as follows:

$$ \mathrm{GS}\ 0:\kern0.5em {G}_i=0\kern0.5em \mathrm{for}\ \mathrm{all}\ \mathrm{i}\ \mathrm{and}\ \mathrm{GS}\ 1:\kern0.5em {G}_2-{G}_1=1/3 $$

For detecting the group×peptide interaction effect, we set (*G* × *P*)_*i*, *k*_, as follows:

$$ {\left(G\times P\right)}_{i,k}=\left({e}_{GP}-\frac{2\left(k-1\right)}{K-1}{e}_{GP}\right){\left(-1\right)}^i\kern0.5em \mathrm{for}\kern0.5em i=1,2\kern0.5em \mathrm{and}\kern0.5em k=1,\dots, K,\kern0.5em \mathrm{where}\kern0.5em {e}_{GP}>0\kern0.5em \mathrm{and}\kern0.5em {e}_{GP}=\sqrt{3\frac{K-1}{K+1}}{\sigma}_{GP}. $$

Here, using *σ*_*GP*_ values determined from the squared average of the interaction effect, we considered three interaction effect scenarios (ISs) for *σ*_*GP*_, as follows:

$$ \mathrm{IS}\ 1:\kern0.5em {\sigma}_{GP}^2=0.05\kern0.5em \mathrm{and}\kern0.5em \mathrm{IS}\ 2:\kern0.5em {\sigma}_{GP}^2=0.1 $$

### Materials

To identify candidate serum hepatocellular carcinoma (HCC) biomarkers for prognosis and response to the tyrosine kinase inhibitor sorafenib, data from 115 HCC patients who had undergone continuous administration of sorafenib for more than 6 weeks, were collected from Seoul National University Hospital, as part of an ongoing study between May 2013 and August 2014. HCC was diagnosed by histological or radiological evaluation, with reference to the American Association for the Study of Liver Diseases (AASLD) [31] or the European Association for the Study of the Liver (EASL) [32] guidelines. All procedures/analyses were approved by the Seoul National University Hospital Institutional Review Board (IRB protocol No. 0506–150-005). Sorafenib response was evaluated using the modified response evaluation criteria in solid tumors (mRECIST) [28], using independent radiologic assessments. Patients with complete response, partial response, and stable disease were categorized as responders, while those with progressive disease were categorized as non-responders.

Toward these objectives, a total of 115 serum samples were randomly separated into two batches: the 1st batch consisted of 65 samples and the 2nd batch consisted of 50 samples. Of those, the total number of responders was 40, and the number of non-responders was 75. One hundred twenty-four candidate protein biomarkers, known hepatic disease-associated proteins, were chosen from 50,265 proteins, based on the LiverAtlas database [27]. One to seven peptides, comprised within each protein, were measured, and counted (Table 2).

**Table 2:** The number of proteins of each number of peptides

The number of peptides	1	2	3	4	5	6	7
The number of proteins	48	57	12	5	0	1	1

Since there were some cases in which LMM(FF), LMM(FR), and L did not preserve type I error in our simulation studies, we applied LMM(RF), LMM(RR), W, *W*_1_, *W*_*S*_ and *S*_*VC*_ to analyze the MRM data. Quantile normalization, provided within the MSstats package [20], was employed for preprocessing. To adjust the mean differences between the two batches, batch indicators were included in models (1), (3), and (8) for MRM data analysis.

## Results

### Simulation results

The type I error and the empirical power were estimated as the proportion of *p*-values under 0.05, out of 1000 repetitions. The type I error rates of LMMs and LR-SAM are summarized in Table 3. The true type of subject effect more strongly affected the type I error rate of LMMs, as compared to the run effect. Analogously, when the true subject effect was fixed, the four LMMs controlled the type I errors. Among the five LR-SAM tests, L could not control the type I error rate when the sample size was 20, while the other four LR-SAM tests could. When the sample sizes were 50 and 100, all LR-SAM tests controlled type I error. When the true subject effect was random, LMM(FF) and LMM(FR) could not control type I error, whereas LMM(RF) and LMM(RR) could. L did not control the type I error rate when the sample sizes were 20 and 50, while the other four LR-SAM tests did. The AIC value of LMM(FF) tended to be the smallest among the four LMMs, under any conditions. Thus, LMM(FF) was most frequently selected as the best LMM.

**Table 3:** Estimated type I error of LMMs and LR-SAM methods

True subject type	True run type	Sample size	Model									
			LMM					LR-SAM				
			Best	(FF)	(FR)	(RF)	(RR)	*L*	*W*	*W*_1_	*W*_*S*_	*S*_*VC*_
Fixed	Fixed	20	0.053	0.054	0.037	0.006	0.002	0.093	0	0.002	0.002	0.019
		50	0.038	0.038	0.033	0.006	0.001	0.044	0.002	0	0.004	0.012
		100	0.048	0.048	0.03	0.003	0	0.035	0.014	0.001	0.001	0.012
	Random	20	0.052	0.052	0.055	0.004	0.002	0.082	0	0.002	0.001	0.014
		50	0.054	0.054	0.049	0.01	0.005	0.055	0.007	0.008	0.006	0.017
		100	0.032	0.032	0.033	0.003	0.001	0.043	0.025	0.003	0.003	0.024
Random	Fixed	20	0.152	0.153	0.211	0.029	0.031	0.107	0	0.008	0.015	0.039
		50	0.178	0.179	0.26	0.06	0.055	0.065	0.014	0.042	0.053	0.05
		100	0.148	0.148	0.204	0.041	0.029	0.06	0.033	0.033	0.039	0.04
	Random	20	0.157	0.159	0.184	0.046	0.053	0.114	0	0.008	0.027	0.053
		50	0.168	0.168	0.195	0.055	0.06	0.068	0.019	0.039	0.047	0.049
		100	0.16	0.16	0.17	0.045	0.04	0.06	0.031	0.034	0.039	0.054

Some LR-SAM tests showed increased power as the interaction effects became large. When there was only an interaction effect, without group effects, the power of the LMMs *W*_1_, and *W*_*S*_ did not increase, as shown in Table 4. As the sample size and the interaction effect became large, W and *S*_*VC*_ showed increased power. Moreover, *S*_*VC*_ provided higher power than W, when the sample size was 50, while W provided higher power than *S*_*VC*_, when the sample size was 100.

**Table 4:** Estimated power of LMMs and LR-SAM methods for GS 0. Bolded number indicates power of methods were more than 0.8

Interaction scenario	True subject type	True run type	Sample size	Model									
				LMM					LR-SAM				
				Best	(FF)	(FR)	(RF)	(RR)	*L*	*W*	*W*_1_	*W*_*S*_	*S*_*VC*_
IS1	Fixed	Fixed	20	0.049	0.05	0.03	0.002	0.001	0.344	0	0	0.001	0.135
			50	0.048	0.048	0.029	0.002	0	0.692	0.385	0.008	0.002	0.506
			100	0.053	0.053	0.041	0.007	0.001	**0.953**	**0.926**	0.007	0.007	**0.911**
		Random	20	0.051	0.052	0.066	0.004	0.002	0.339	0.001	0.002	0.002	0.14
			50	0.047	0.047	0.042	0.002	0.001	0.691	0.397	0.007	0.002	0.538
			100	0.045	0.045	0.043	0.003	0.003	**0.953**	**0.93**	0.008	0.003	**0.914**
	Random	Fixed	20	0.147	0.149	0.198	0.051	0.046	0.402	0.001	0.009	0.027	0.213
			50	0.177	0.177	0.234	0.048	0.042	0.745	0.415	0.025	0.04	0.593
			100	0.157	0.157	0.201	0.054	0.049	**0.95**	**0.923**	0.037	0.051	**0.917**
		Random	20	0.158	0.159	0.19	0.052	0.06	0.415	0	0.003	0.026	0.216
			50	0.148	0.15	0.168	0.04	0.046	0.726	0.455	0.027	0.034	0.602
			100	0.164	0.165	0.171	0.04	0.043	**0.975**	**0.948**	0.026	0.037	**0.932**
IS2	Fixed	Fixed	20	0.054	0.055	0.04	0.005	0	0.611	0.001	0.001	0.001	0.353
			50	0.051	0.051	0.034	0.009	0.002	**0.945**	0.787	0.018	0.004	**0.896**
			100	0.045	0.045	0.036	0.007	0.001	**1**	**1**	0.016	0.006	**1**
		Random	20	0.044	0.046	0.058	0.004	0.006	0.606	0	0.002	0.001	0.354
			50	0.045	0.046	0.046	0.001	0.001	**0.953**	0.8	0.014	0.001	**0.907**
			100	0.056	0.056	0.062	0.01	0.009	**0.999**	**0.998**	0.012	0.009	**0.998**
	Random	Fixed	20	0.145	0.147	0.2	0.055	0.046	0.592	0	0.005	0.033	0.44
			50	0.143	0.145	0.2	0.034	0.028	**0.957**	**0.801**	0.029	0.026	**0.91**
			100	0.166	0.166	0.223	0.049	0.04	**1**	**1**	0.043	0.045	**1**
		Random	20	0.162	0.164	0.188	0.048	0.057	0.639	0	0.001	0.021	0.443
			50	0.144	0.145	0.177	0.04	0.044	**0.949**	**0.801**	0.02	0.031	**0.901**
			100	0.17	0.17	0.206	0.053	0.06	**0.999**	**0.999**	0.032	0.048	**0.999**

The size of the group effect showed large effects on all LMMs and LR-SAM methods. The estimated powers of the five LMMs, and the LR-SAM methods for GS 1, are shown in Table 5. The powers of L, W, *W*_1_, and *S*_*VC*_ were all affected by the size of the interaction effect, when the group effect scenario was GS 1, while those of the five LMMs did not. As the interaction effect increased, the powers of L, W, and *S*_*VC*_ increased, while the powers of four of the LMMs, and *W*_*S*_, did not, and the power of the *W*_1_ method even decreased. When the interaction scenario was IS 1, the powers of the L and *S*_*VC*_ methods were higher than those of the LMMs, for a sample size of 100. For the IS 2 scenario, once the sample size exceeded 50, the L and *S*_*VC*_ methods provided higher powers than those of the LMMs.

**Table 5:** Estimated power of LMMs and LR-SAM methods for GS 1. Bolded number indicates power of methods were more than 0.8

Interaction scenario	True subject type	True run type	Sample size	Model									
				LMM					LR-SAM				
				Best	(FF)	(FR)	(RF)	(RR)	*L*	*W*	*W*_1_	*W*_*S*_	*S*_*VC*_
IS1	Fixed	Fixed	20	0.296	0.301	0.459	0.077	0.044	0.417	0	0.01	0.042	0.258
			50	0.627	0.63	**0.85**	0.333	0.216	0.798	0.548	0.14	0.291	0.756
			100	**0.916**	**0.917**	**0.994**	0.709	0.632	**0.996**	**0.984**	0.424	0.694	**0.995**
		Random	20	0.302	0.303	0.39	0.085	0.093	0.42	0	0.007	0.034	0.253
			50	0.642	0.646	0.715	0.321	0.336	**0.805**	0.534	0.154	0.288	0.775
			100	**0.921**	**0.923**	**0.958**	0.681	0.753	**0.99**	**0.981**	0.405	0.668	**0.99**
	Random	Fixed	20	0.351	0.35	0.472	0.152	0.159	0.472	0.001	0.016	0.095	0.304
			50	0.626	0.628	0.782	0.384	0.403	**0.825**	0.574	0.218	0.359	0.782
			100	**0.85**	**0.851**	**0.928**	0.659	0.705	**0.985**	**0.976**	0.457	0.652	**0.983**
		Random	20	0.379	0.381	0.43	0.176	0.186	0.488	0	0.024	0.106	0.348
			50	0.613	0.615	0.674	0.366	0.389	**0.812**	0.552	0.19	0.342	0.775
			100	**0.834**	**0.835**	**0.873**	0.655	0.679	**0.989**	**0.981**	0.447	0.639	**0.98**
IS2	Fixed	Fixed	20	0.328	0.331	0.482	0.083	0.039	0.662	0	0.005	0.042	0.503
			50	0.619	0.62	**0.865**	0.298	0.199	**0.978**	**0.87**	0.095	0.268	**0.966**
			100	**0.889**	**0.889**	**0.996**	0.696	0.621	**1**	**1**	0.279	0.676	**1**
		Random	20	0.288	0.29	0.382	0.067	0.074	0.649	0	0.006	0.023	0.466
			50	0.648	0.652	0.735	0.339	0.363	**0.979**	**0.888**	0.112	0.305	**0.97**
			100	**0.921**	**0.921**	**0.96**	0.703	0.748	**1**	**1**	0.288	0.685	**1**
	Random	Fixed	20	0.36	0.363	0.493	0.148	0.152	0.683	0.001	0.009	0.093	0.524
			50	0.599	0.602	0.76	0.363	0.377	**0.982**	**0.878**	0.137	0.334	**0.961**
			100	**0.861**	**0.861**	**0.93**	0.674	0.715	**1**	**1**	0.321	0.66	**1**
		Random	20	0.384	0.386	0.462	0.175	0.191	0.682	0	0.007	0.113	0.523
			50	0.583	0.586	0.637	0.357	0.384	**0.968**	**0.869**	0.123	0.323	**0.952**
			100	**0.836**	**0.836**	**0.874**	0.655	0.698	**1**	**1**	0.33	0.644	**1**

Collectively, as the size of group effects became large, all methods provided increased power. As the interaction effect became large, only the L, W, and *S*_*VC*_ methods provided increased power.

### HCC data analysis

**Fig. 1:**
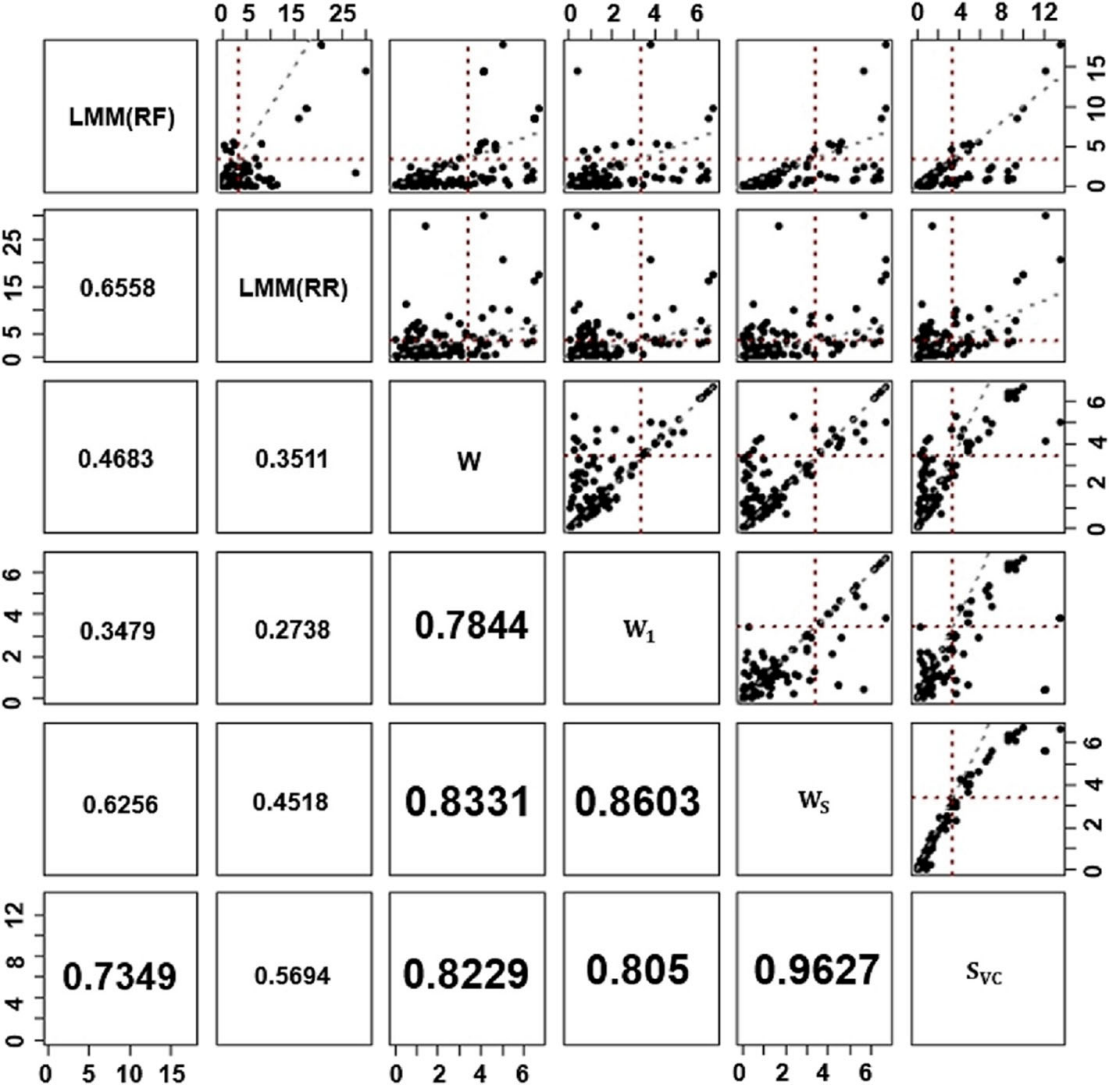
Pairwise scatter plot of –log10-transformed *p*-values from the LMM(RF), LMM(RR), W, W_**1**_, W_S_ and S_VC_ models based on multiple reaction monitoring (MRM) data. Vertical and horizontal dashed red lines represent Bonferroni-corrected significance levels, −log_10_(0.05/124). Diagonally dashed gray line represents one to one slope

Using the approaches described above, the maximum –log10-transformed *p*-values of the linear mixed models (LMMs) were higher than those of the LR-SAM methods. However, the correlation of the transformed p-values of LMM(RF) and LMM(RR) was lower than that of LR-SAM methods, as shown in Fig. 1. The correlation between LMM(RF) and LMM(RR) was 0.6558, while the minimum correlation among the LR-SAM methods was 0.784. The correlation between *W*_*S*_ and *S*_*VC*_ was the highest (0.9627), among the LR-SAM methods.

The lowest p-values of LMM(RF) and LMM(RR) were lower than those of the LR-SAM methods, valued at 1.87 × 10^−18^ and 1.09 × 10^−30^, respectively. The lowest p-value of W, *W*_1_, and *W*_*S*_ was 2 × 10^−7^, and the lowest p-value of *S*_*VC*_ was 3.49 × 10^−14^ (Table 6). Although the lowest p-values of the LR-SAM methods were less significant than those of the LMMs, and for many proteins, LR-SAM methods provided higher significance results (Fig. 1).

**Table 6:** List of proteins and their *p*-values simultaneously identified by LMM or LR-SAM methods

Protein	Models						# of peptide	Simultaneously Identified model
	LMM(RF)	LMM(RR)	*W*	*W*_1_	*W*_*S*_	*S*_*VC*_		
IGJ	2.E-18	3.E-21	1.E-05	1.E-04	2.E-07	3.E-14	3	All
IGHG3	2.E-10	3.E-18	2.E-07	2.E-07	2.E-07	9.E-11	1	All
IGHG1	3.E-09	8.E-17	3.E-07	3.E-07	3.E-07	3.E-10	1	All
GPX3	5.E-06	5.E-09	9.E-05	9.E-05	9.E-05	1.E-05	1	All
FBLN1	1.E-01	7.E-11	3.E-05	1.E-05	5.E-06	2.E-07	2	LR-SAM
C163A	3.E-03	2.E-08	7.E-07	7.E-07	7.E-07	4.E-10	1	LR-SAM
ISLR	2.E-01	6.E-04	4.E-07	4.E-07	4.E-07	1.E-09	1	LR-SAM
FCG3A	8.E-02	8.E-04	6.E-07	6.E-07	6.E-07	3.E-09	1	LR-SAM
QSOX1	2.E-01	2.E-03	8.E-07	8.E-07	8.E-07	3.E-09	1	LR-SAM
FBLN3	2.E-02	2.E-06	4.E-07	4.E-07	4.E-07	3.E-09	1	LR-SAM
SHBG	4.E-03	1.E-04	1.E-05	5.E-05	2.E-06	9.E-08	2	LR-SAM
LG3BP	1.E-02	4.E-06	3.E-05	5.E-06	5.E-06	2.E-07	2	LR-SAM
CATB	2.E-01	2.E-03	7.E-06	7.E-06	7.E-06	3.E-07	1	LR-SAM
HPT	6.E-06	3.E-03	1.E-04	2.E-05	3.E-05	7.E-06	2	LR-SAM
POSTN	9.E-02	3.E-04	1.E-04	1.E-04	1.E-04	1.E-05	1	LR-SAM
SODE	4.E-02	7.E-05	2.E-04	2.E-04	2.E-04	2.E-05	1	LR-SAM
1433S	1.E-01	8.E-04	1.E-04	1.E-04	1.E-04	2.E-05	1	LR-SAM
FSTL1	2.E-01	2.E-03	5.E-05	5.E-05	5.E-05	7.E-05	1	LR-SAM
CD5L	3.E-15	1.E-30	8.E-05	4.E-01	2.E-06	9.E-13	3	LMM
APOA4	3.E-04	1.E-07	1.E-03	1.E-01	7.E-04	2.E-04	2	LMM

**Fig. 2:**
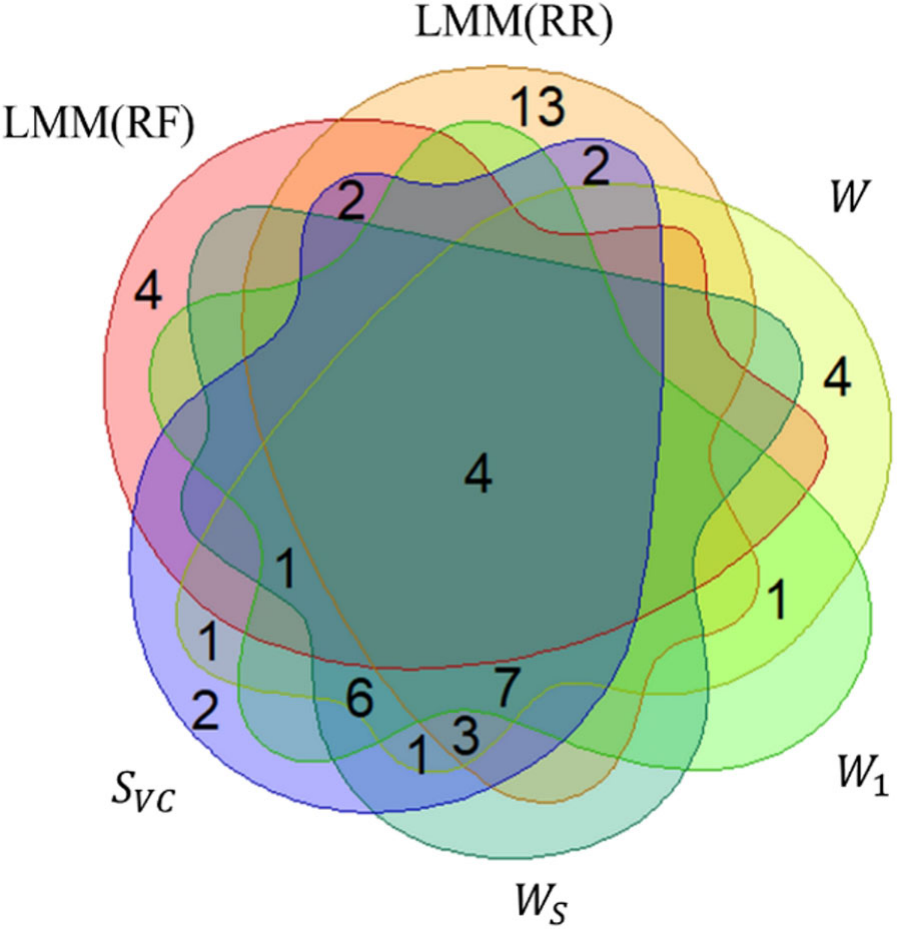
Venn diagram of proteins identified from LMM (RF, RR) and LR-SAM (W, W_1_, W_S_ and S_VC_), under a Bonferroni correction significance level of −log_10_(0.05/124)

The LMM(RF) and LMM(RR) methods identified 11 and 37 proteins, respectively. There were six (14.29%) proteins simultaneously identified by LMM(RF) and LMM(RR). For LR-SAM methods, W, *W*_1_, *W*_*S*_, and *S*_*VC*_ identified 28, 19, 22, and 29 proteins, respectively. Among these proteins 18 (52.94%) were simultaneously identified by four methods. Finally, there were four (7.84%) proteins identified by all six methods of LMM and LR-SAM (Fig. 2).

All LMMs and LR-SAM methods simultaneously provided significance results for the presence of the proteins GPX3, IGHG1, IGHG3 and IGJ. Among these, IGJ (immunoglobulin J chain, linker protein for immunoglobulin alpha and mu) was previously reported as having a significant difference of expression in HCC tumors [33], while GPX3 (glutathione peroxidase 3) was reported as a tumor suppressor in HCC [34].

There were 14 proteins that the LR-SAM methods, but not the LMMs, simultaneously provided significance results of expression (Table 6). Among those 14, seven were previously reported, including FBLN1 (Fibulin-1), a tumor suppressor gene in HCC [35], SHBG (sex hormone-binding globulin), a prediagnostic risk marker for HCC [36], and LG3BP (galectin-3-binding protein), a potential marker in six cancer types, including HCC, lymphoma, NPC (nasopharyngeal carcinoma), CRC (colorectal carcinoma), and oral cancers [37]. Additionally, CATB (Cathepsin B) was previously reported as a potential candidate cancer biomarker in HCC [38], HPT (haptoglobin) was reported to associate with tumor progression in HCC [39], while POSTN (periostin) was reported as a marker for malignant transformation of hepatocytes [40]. Similarly, 14–3-3S (14–3-3 protein sigma) was reported as downregulated in HCC [41].

There were two proteins that LMMs simultaneously provided a significance result, while LR-SAM methods did not. Resultant *p*-values for the simultaneously identified 20 proteins are shown in Table 6. Two proteins that demonstrated significance by LMM(RF) and LMM(RR) were also previously reported: CD5L (CD5 antigen-like), reported as differentially expressed in hepatitis C patients [42]: and APOA4, (apolipoprotein A-IV), reported as misexpressed in liver metabolic disorders [43].

## Discussion

We examined the performance of LMMs and LR-SAM methods, through extensive simulation studies. When the true subject effect was random, LMM(FF) and LMM(FR) did not preserve type I error (Table 3). We also applied Akaike information criterion (AIC), for model selection, to check whether or not the best-performing LMM preserved type I error. However, our empirical study showed that LMM(FF) had the smallest AIC value for most simulation settings, which made it difficult to use AIC as a model selection criterion. On the other hand, LR-SAM methods, except L, well preserved type I error under any type of subject and run effects.

Since the hypothesis (2) did not consider the interaction effect, the power of the LMM approach was not affected under any size of the interaction effect. However, when the interaction effect (without the group effect) was considered, hypothesis (2) could not perform well, as we observed through our simulation studies. On the other hand, testing hypotheses (8) with W and (10) with *S*_*VC*_, changes under any size group effect and interaction effect were detectable. Additionally, LR-SAM methods did not provide higher power than LMMs, when there were no or only weak interaction effects.

Although LMM provided more protein significance results than LR-SAM methods, the proteins identified by the LMM(RF) and LMM(RR) approaches were very different from each other. On the other hand, LR-SAM methods provided more consistent significance results than those of the LMMs. Moreover, LR-SAM methods provided greater significance results than those identified by LMM(RF), for most proteins, and several proteins were identified only by LR-SAM methods. However, there was a still performance difference between the choices of fixed and random effect models in LR-SAM methods, as was in the LMM. This difference was caused by different testing hypotheses between W and S_VC_ in LR-SAM. In addition, note that S_VC_ allows the heterogenous effects of peptides, while W does not.

## Conclusion

LMMs have been widely used to identify proteins with significantly altered abundance, in distinct disease states, based on MRM assays [39]. However, we found that LMM approaches provide inconsistent significance results for the same MRM data, depending on which effects are treated as random or fixed by simulation results. It is a well-known property of LMMs that the variance of model parameters are underestimated, when the fixed effect model is fitted but the true effects are random, and vice versa [44]. As a result, the protein significance results of LMMs may vary, depending on whether the true effect is random or fixed, and we also observed this phenomenon, as shown in Fig. 1 and Table 6. Thus, it is highly important to correctly specify the effect as random or fixed.

In this paper, we propose a new logistic regression-based method for Significance Analysis of Multiple Reaction Monitoring (LR-SAM). Unlike LMMs, our LR-SAM approach uses a much smaller number of parameters. Moreover, our LR-SAM does not require inclusion of all the effects related to the run. Accordingly, our model does not need to specify run effects as random or fixed.

In simulation study, our LR-SAM preserved type I error when the true subject effect was random, while LMM(FF) and LMM(FR) did not. In real data analysis, although LMM provided more protein significance results than LR-SAM methods, LR-SAM methods provided more consistent significance results than those of the LMMs. Thus, our proposed method, LR-SAM could give more reliable results than previous protein studies.

## Abbreviations

AASLD: American Association for the Study of Liver Diseases
AIC: Akaike information criterion
ANOVA: Analysis of variance
EASL: European Association for the Study of the Liver
ELISA: Enzyme-linked immunosorbent assay
GS: Group effect scenarios
HCC: Hepatocellular carcinoma
IS: Interaction effect scenarios
LMM: Linear mixed model
mRECIST: Modified response evaluation criteria in solid tumors
MRM: Multiple reaction monitoring
